# Genetic and Environmental Factors and Cardiovascular Disease Risk in Adolescents

**DOI:** 10.1001/jamanetworkopen.2023.43947

**Published:** 2023-11-17

**Authors:** Marcel Ballin, Martin Neovius, Francisco B. Ortega, Pontus Henriksson, Anna Nordström, Daniel Berglind, Peter Nordström, Viktor H. Ahlqvist

**Affiliations:** 1 Centre for Epidemiology and Community Medicine, Region Stockholm, Stockholm, Sweden; 2 Department of Public Health and Caring Sciences, Clinical Geriatrics, Uppsala University, Uppsala, Sweden; 3 Department of Medicine, Clinical Epidemiology Division, Karolinska Institutet, Stockholm, Sweden; 4 Department of Physical Education and Sports, Faculty of Sport Sciences, Sport and Health University Research Institute, University of Granada, Granada, Spain; 5 Faculty of Sport and Health Sciences, University of Jyväskylä, Jyväskylä, Finland; 6 CIBER de Fisiopatología de la Obesidad y Nutrición, Instituto de Salud Carlos III, Granada, Spain; 7 Department of Health, Medicine and Caring Sciences, Linköping University, Linköping, Sweden; 8 Rehabilitation and Pain Centre, Uppsala University Hospital, Uppsala, Sweden; 9 School of Sport Sciences, UiT the Arctic University of Norway, Tromsø, Norway; 10 Department of Health Sciences, The Swedish Winter Sport Research Centre, Mid Sweden University, Östersund, Sweden; 11 Department of Global Public Health, Karolinska Institutet, Stockholm, Sweden

## Abstract

**Importance:**

Cardiovascular risk factors in youth have been associated with future cardiovascular disease (CVD), but conventional observational studies are vulnerable to genetic and environmental confounding.

**Objective:**

To examine the role of genetic and environmental factors shared by full siblings in the association of adolescent cardiovascular risk factors with future CVD.

**Design, Setting, and Participants:**

This is a nationwide cohort study with full sibling comparisons. All men who underwent mandatory military conscription examinations in Sweden between 1972 and 1995 were followed up until December 31, 2016. Data analysis was performed from May 1 to November 10, 2022.

**Exposures:**

Body mass index (BMI), cardiorespiratory fitness, blood pressure, handgrip strength, and a combined risk *z* score in late adolescence.

**Main Outcomes and Measures:**

The primary outcome was fatal or nonfatal CVD, as recorded in the National Inpatient Register or the Cause of Death Register before 2017.

**Results:**

A total of 1 138 833 men (mean [SD] age, 18.3 [0.8] years), of whom 463 995 were full brothers, were followed up for a median (IQR) of 32.1 (26.7-37.7) years, during which 48 606 experienced a CVD outcome (18 598 among full brothers). All risk factors were associated with CVD, but the effect of controlling for unobserved genetic and environmental factors shared by full siblings varied. In the sibling analysis, hazard ratios for CVD (top vs bottom decile) were 2.10 (95% CI, 1.90-2.32) for BMI, 0.77 (95% CI, 0.68-0.88) for cardiorespiratory fitness, 1.45 (95% CI, 1.32-1.60) for systolic blood pressure, 0.90 (95% CI, 0.82-0.99) for handgrip strength, and 2.19 (95% CI, 1.96-2.46) for the combined *z* score. The percentage attenuation in these hazard ratios in the sibling vs total cohort analysis ranged from 1.1% for handgrip strength to 40.0% for cardiorespiratory fitness. Consequently, in the sibling analysis, the difference in cumulative CVD incidence at age 60 years (top vs bottom decile) was 7.2% (95% CI, 5.9%-8.6%) for BMI and 1.8% (95% CI, 1.0%-2.5%) for cardiorespiratory fitness. Similarly, in the sibling analysis, hypothetically shifting everyone in the worst deciles of BMI to the middle decile would prevent 14.9% of CVD at age 60 years, whereas the corresponding number for cardiorespiratory fitness was 5.3%.

**Conclusions and Relevance:**

In this Swedish national cohort study, cardiovascular risk factors in late adolescence, especially a high BMI, were important targets for CVD prevention, independently of unobserved genetic and environmental factors shared by full siblings. However, the role of adolescent cardiorespiratory fitness in CVD may have been overstated by conventional observational studies.

## Key Points

**Question:**

What is the role of genetic and shared environmental factors in the association of adolescent cardiovascular risk factors with future cardiovascular disease (CVD)?

**Findings:**

This nationwide cohort study of more than 1 million men found that body mass index, blood pressure, cardiorespiratory fitness, and handgrip strength in late adolescence were associated with CVD in adulthood, even after controlling for unobserved genetic and environmental factors shared by full brothers, although the degree of attenuation from adjusting for familial factors varied by risk factor. A high body mass index remained the most important factor, whereas the benefits of cardiorespiratory fitness were markedly attenuated.

**Meaning:**

These findings suggest that for effective CVD prevention, halting and reversing the obesity epidemic should be prioritized.

## Teaser

This cohort study examines the role of genetic and environmental factors shared by full siblings in the association of cardiovascular risk factors during adolescence with future cardiovascular disease.

## Introduction

The global burden of cardiovascular diseases (CVDs) remains substantial and is increasing in some locations, mainly owing to population growth and aging, but possibly also reflecting negative trends in modifiable risk factors.^1,2^ Interventions targeting such factors are, therefore, considered important in CVD prevention,^3^ with emphasis on initiatives targeting young people, which is when the foundation for lifelong habits is set.

Although youth risk factors for subclinical CVD in adulthood have been identified, evidence regarding clinical outcomes is sparser.^4^ A few studies have shown that cardiovascular risk factor clustering in childhood^5^ and individual factors, such as body mass index (BMI), cardiorespiratory fitness, blood pressure, and muscular strength in late adolescence, are associated with later cardiovascular outcomes.^6,7,8,9,10,11^ However, a sizeable proportion of the variation in these risk factors^12,13,14^ and in CVD^15,16^ is attributable to heritable and environmental influences. Previous studies may, therefore, have been confounded by genetic and environmental factors, potentially overestimating the presumed benefit of modifying these risk factors in youth.

To strengthen causal inference, it is possible to study discordant family members wherein all factors shared within families, including unobserved genetic and environmental factors, are inherently controlled for. Unfortunately, such studies are rare. We and others^17,18,19^ have previously found that the associations of adolescent cardiorespiratory fitness and BMI with CVD and all-cause mortality attenuate when controlling for factors shared by twins (possibly irrespective of zygosity). However, twin-based studies are typically limited in power because they rely on a small set of discordant twins. This also makes them susceptible to various biases.^20^ Furthermore, the role of shared familial factors in other cardiovascular risk factors, such as blood pressure and muscular strength, or the combination of risk factors, remains largely unknown. Collectively, the scarcity of studies considering familial factors hinders the current appreciation of the significance of modifying cardiovascular risk factors in youth.

To guide policymaking and public health interventions, it is paramount that we understand the associations of different risk factors with CVD and the magnitude of the expected benefit from modifying them. Therefore, we used Swedish nationwide registers to construct a linked cohort of approximately 1 million young men, of whom approximately half a million were full brothers, to examine the individual and combined roles of late adolescent BMI, cardiorespiratory fitness, blood pressure, and handgrip strength in major CVD later in life, addressing the currently unknown contributions of genetic and environmental factors shared within families to these associations.

## Methods

### Study Design and Databases

We constructed a nationwide cohort study with full sibling comparisons by linking data from Swedish nationwide registers. From the Swedish Military Service Conscription Registry, we obtained an eligible study population with data on adolescent cardiovascular risk factors, all of which were assessed over 2 days according to a standardized procedure.^21^ During the conscription period considered for the present study (1972-1995), conscription around the age of 18 years was mandatory in Sweden, with few exemptions (population coverage was approximately 90%).^21^ Reasons for exemptions included certain functional disabilities, care needs, and residing outside Sweden. Further details on these exemptions are available elsewhere.^21^ We identified all full brothers using data from the Multi-Generation Register.^22^ We collected data on mortality from the National Cause of Death Register, which contains data on deaths of all Swedish citizens since 1951.^23^ We identified all hospitalizations due to CVD from the National Inpatient Register, which was launched in 1964 and became nationwide in 1987.^24^ From the Total Population Register,^22^ we obtained data on the date of emigration. Data were cross-linked using the personal identification number, which is unique for every individual in Sweden.^25^ This study was approved by the Regional Ethical Review Board in Umeå, and the need for informed consent was waived because all data were obtained from registers, in accordance with praxis in Nordic registry research.^26^ The study is reported according to the Strengthening the Reporting of Observational Studies in Epidemiology (STROBE) reporting guidelines.

### Derivation of the Study Population

We identified 1 250 285 men who were conscripted between 1972 and 1995, excluding 86 individuals with preexisting CVD and 2 individuals who emigrated on the day of conscription. We excluded 108 189 individuals (8.7%) with missing data on any of the exposures and 1088 individuals (0.1%) with missing data on birth year or conscription office. In accordance with previous studies based on the same population,^6,17,27^ we also excluded 2175 individuals (0.2%) with extreme values on the exposures (described later).

### Exposures

The exposures were objectively measured BMI (calculated as weight in kilograms divided by height in meters squared), cardiorespiratory fitness (Watt maximum), systolic blood pressure (millimeters of mercury), diastolic blood pressure (millimeters of mercury), and handgrip strength (Newton), all measured using validated tools as described previously.^8,11,21^ We excluded conscripts with extreme values (BMI <15 and >60, cardiorespiratory fitness <100 W maximum, systolic blood pressure <100 and >180 mm Hg, diastolic blood pressure <40 and >100 mm Hg, and handgrip strength <100 N), in accordance with previous studies.^6,17,27^ To shed light on the combined effect of the exposures, we used a method similar to that of a recent study,^5^ where we calculated a combined measure as the unweighted mean across exposure *z* scores. For consistency, we inverted the scale of cardiorespiratory fitness and handgrip strength, and to not give double weight to blood pressure, we instead used the mean arterial pressure ([2 × diastolic + systolic] / 3).

### Outcome

The outcome was major CVD, defined as the composite of hospitalization due to stroke, heart failure, ischemic heart disease, or death from CVD until December 31, 2016. We considered any hospitalization where these outcomes had been recorded by diagnostic codes in either a primary or secondary position (eTable 1 in Supplement 1), and classified a death as death from CVD if it was recorded as the primary cause on the death certificate. The positive predictive value for most diagnoses in the National Inpatient Register is 85% to 95%, including high values for stroke (69%-99%), angina pectoris (95%), heart failure (82%-88%), and myocardial infarction (98%-100%).^24^ The sensitivity is often lower, although still high for diseases such as stroke and myocardial infarction.^24^

### Statistical Analysis

Data analysis was performed from May 1 to November 10, 2022. Participants were censored upon the date of the first outcome event, emigration, death from non-CVD causes, or end of follow-up (December 31, 2016), whichever came first. To estimate hazard ratios (HRs) for CVD according to the level of exposure, we used flexible parametric survival models,^28^ where we estimated the baseline hazard using restricted cubic splines with knots placed at the 5th, 27.5th, 50th, 72.5th, and 95th percentile. Unlike Cox regression, where the baseline hazard cancels out, these models directly model the baseline and, therefore, enable the estimation of absolute measures of association. We considered an estimate to be statistically significant if its 95% CI did not include the null. In the sibling cohort, we extended the flexible parametric model to a marginalized between-within model^29^ with robust (sandwich) SEs, thereby controlling for all factors shared within families (ie, genetic and environmental factors). Between-within models include a term for the exposure and a term for its family average, thereby isolating the individual-level variation (within effect) from the family-level variation (between effect). Further details on these models are available elsewhere.^20,29^ To allow for a nonlinear association, we modeled our exposures both as deciles and using restricted cubic splines with knots placed at the 5th, 35th, 65th, and 95th percentile. All models were adjusted for birth cohort (1950-1952, 1953-1957, 1958-1962, 1963-1967, 1968-1972, and 1973-1977), conscription cohort (1972, 1973-1977, 1978-1982, 1983-1987, 1988-1992, and 1993-1995), and conscription office (6 different sites), as in previous studies.^6,7,8,9,10,11^

We also computed the cumulative failure probability (1 − survival) at 42 years of follow-up specifically and illustrated it across the entire follow-up using the aforementioned models. We hereafter refer to this quantity as the cumulative incidence at age 60 years (because most participants were conscripted at the age of 18 years). Similarly, for an appreciation of the expected public health benefit from intervening upon the risk factors, we calculated the population-attributable fraction (PAF) at age 60 years (after 42 years of follow-up), where we considered a major public health intervention that shifts everyone to the ideal decile of the risk factor (eg, for BMI this would mean shifting all individuals between the 2nd and 10th decile to the 1st decile), or a moderate intervention that shifts everyone who was in either of the worst deciles to the normal decile (eg, for BMI this would mean shifting all individuals between the 6th and 10th deciles to the 5th decile).

Finally, in a sensitivity analysis, we replicated our cohort analysis in the sibling cohort to elucidate whether any observed differences were due to selection bias rather than control for shared familial factors. All analyses were conducted using Stata statistical software version 15.1 (StataCorp).

## Results

### Baseline Characteristics

The total cohort included 1 138 833 conscripts (91.1% retained), 92.2% of whom were conscripted at the age of 18 years. For the sibling analysis, we identified all conscripts that were full brothers, resulting in the inclusion of 463 995 full brothers from 213 161 families (Table 1). The mean (SD) age at conscription was 18.3 (0.8) years, and the median (IQR) birth year was 1965 (1959-1970). The median (IQR) follow-up was 32.1 (26.7-37.7) years, with a maximum of 44.4 years. The numbers censored were 71 728 men (6.3%) for emigration, 34 028 men (3.0%) for death from non-CVD causes, and 984 471 men (86.5%) at the end of follow-up. During follow-up, 48 606 conscripts (4.3%) experienced a CVD event (43 005 nonfatal and 5601 fatal events). The median (IQR) age at the CVD event was 50.0 (44.3-55.1) years, with a maximum observed age of 70.7 years. In the sibling cohort, there were 18 598 (4.0%) CVD events (16 579 nonfatal and 2016 fatal events), with baseline characteristics similar to that of the total cohort.

**Table 1.** Baseline Characteristics in the Total Cohort and in the Sibling Cohort

Characteristic	Total cohort (N = 1 138 833)	Sibling cohort (n = 463 995)
Birth year, median (IQR)	1965 (1959 to 1970)	1965 (1960 to 1970)
Age at conscription, mean (SD), y	18.3 (0.8)	18.3 (0.7)
Body mass index^a^		
Mean (SD)	21.7 (2.8)	21.8 (2.8)
Median (range)	21.3 (15.0 to 54.7)	21.3 (15.0 to 51.3)
Cardiorespiratory fitness, W maximum		
Mean (SD)	274 (52)	274 (52)
Median (range)	270 (100 to 999)	270 (100 to 999)
Systolic blood pressure, mm Hg		
Mean (SD)	129 (11)	129 (11)
Median (range)	128 (100 to 180)	128 (100 to 180)
Diastolic blood pressure, mm Hg		
Mean (SD)	67 (10)	66 (10)
Median (range)	68 (40 to 100)	66 (40 to 100)
Handgrip strength, N		
Mean (SD)	616 (98)	617 (98)
Median (range)	610 (100 to 999)	610 (100 to 999)
Combined risk, *z* score^b^		
Mean (SD)	0.0 (0.5)	0.0 (0.5)
Median (range)	0.0 (−3.6 to 4.2)	0.0 (−3.6 to 3.7)

^a^ Body mass index is calculated as weight in kilograms divided by height in meters squared.

^b^ Refers to the unweighted mean across exposure *z* scores (see the Methods section).

### HRs for CVD by Adolescent Risk Factors

**Figure 1.**
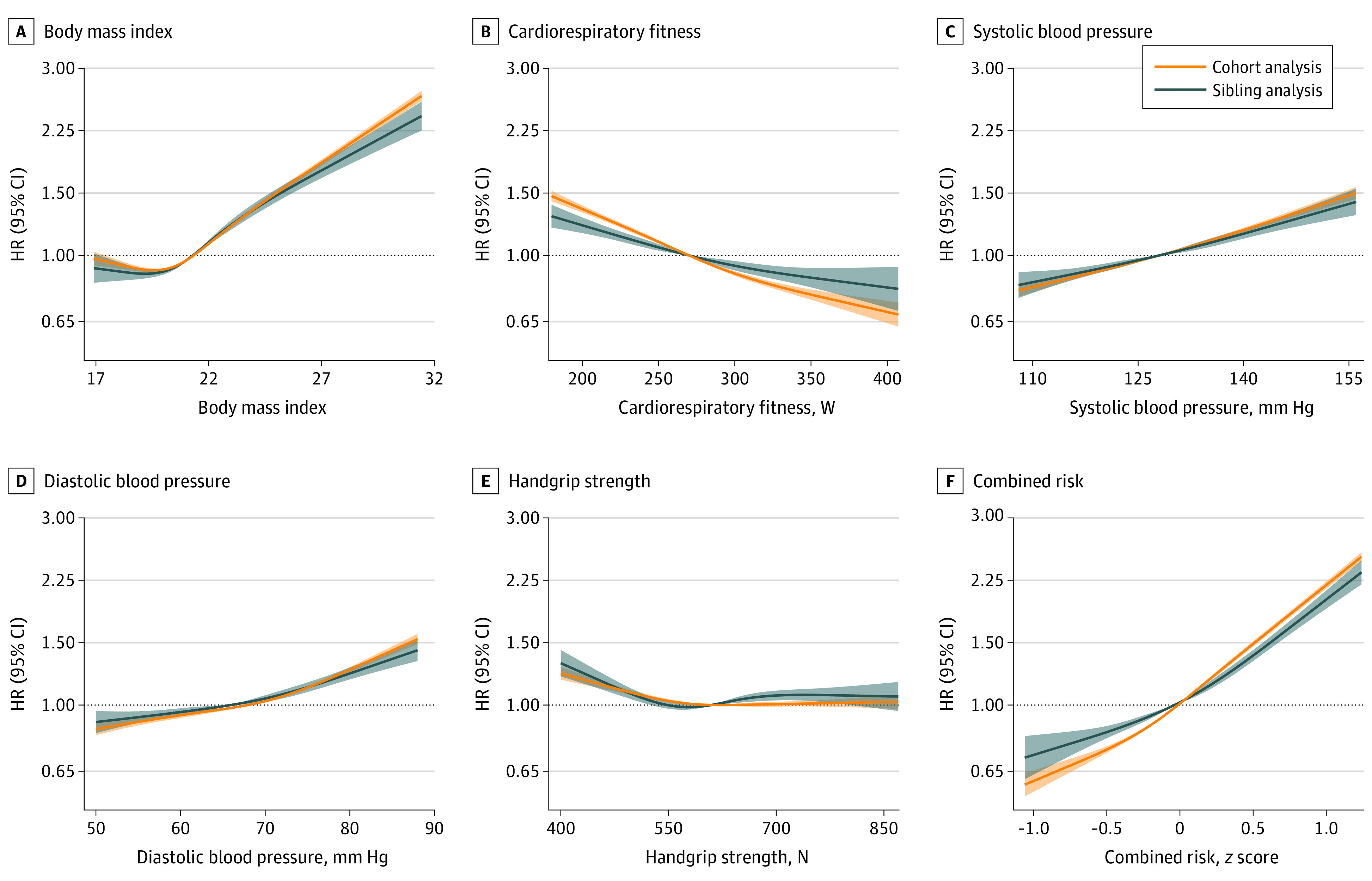
Hazard Ratios (HRs) for Cardiovascular Disease by Risk Factors in the Total Cohort and Sibling Cohort Estimates were obtained using flexible parametric survival models, by which the associations across the restricted cubic splines of exposures were computed with the median value as the referent. Shaded areas denote 95% CIs. The models were adjusted for birth cohort, conscription cohort, and conscription office. For graphical purposes, the x-axis was limited to span from the first to the 99th percentile of the exposure distribution. The combined risk *z* score represents the unweighted mean across exposure *z* scores.

All risk factors were associated with CVD, but the effect of controlling for factors shared by full brothers varied among the risk factors (Figure 1 and Table 2). For BMI, the HR comparing the top vs bottom decile was 2.10 (95% CI, 1.90-2.32), after 6.7% attenuation compared with the total cohort analysis. As for cardiorespiratory fitness, there was 15.6% attenuation comparing the middle vs bottom decile (HR, 0.89; 95% CI, 0.82-0.97), and 40.0% attenuation comparing the top vs bottom decile (HR, 0.77; 95% CI, 0.68-0.88). Systolic and diastolic blood pressure remained associated with CVD to a similar magnitude in the sibling analysis (top decile, HR, 1.45; 95% CI, 1.32-1.60; bottom decile, HR, 1.45; 95% CI, 1.31-1.61), after moderate attenuation (13.2% and 14.7%, respectively). Associations for handgrip strength were attenuated by 1.1%, although it was the risk factor with the smallest association with CVD (top vs bottom decile HR, 0.90; 95% CI, 0.82-0.99). For the combined risk *z* score, the HR comparing the top vs bottom decile was 2.19 (95% CI, 1.96-2.46), after 29.8% attenuation. There were no material differences between the total cohort analysis and the same analysis applied to the sibling cohort, suggesting that differences in estimation between cohort analysis and sibling analysis are not due to differences in the study population (eTable 2 in Supplement 1).

**Table 2.** HRs and Absolute Differences in Incidence for Major Cardiovascular Disease at Age 60 Years by Tenths (Deciles) of the Individual Risk Factors and the Combined Risk *z* Score

Risk factor value per decile, median (range)	Total cohort analysis (N = 1 138 833)			Sibling analysis (n = 463 995)			Change in HR for sibling vs cohort analysis, %^a^
	Cases, No./total No.	HR (95% CI)	Absolute incidence difference at age 60 y, % (95% CI)	Cases, No./total No.	HR (95% CI)	Absolute incidence difference at age 60 y, % (95% CI)	
Body mass index^b^							
18.1 (15.0 to 18.7)	4667/114 967	Reference	Reference	1726/46 851	Reference	Reference	Reference
19.2 (18.7 to 19.6)	4374/115 736	0.98 (0.94 to 1.02)	−0.2 (−0.5 to 0.2)	1731/47 772	1.06 (0.97 to 1.16)	0.5 (−0.3 to 1.2)	8.2
19.9 (19.6 to 20.2)	4181/110 981	1.00 (0.96 to 1.05)	0 (−0.3 to 0.3)	1601/46 119	1.02 (0.93 to 1.12)	0.2 (−0.6 to 0.9)	2.0
20.5 (20.2 to 20.8)	4294/116 307	1.01 (0.97 to 1.05)	0 (−0.3 to 0.4)	1646/48 369	1.06 (0.97 to 1.17)	0.5 (−0.3 to 1.3)	5.0
21.1 (20.8 to 21.3)	4287/113 037	1.06 (1.02 to 1.10)	0.4 (0.1 to 0.8)	1686/46 907	1.19 (1.08 to 1.31)	1.5 (0.6 to 2.3)	12.3
21.6 (21.3 to 21.9)	4225/112 486	1.07 (1.02 to 1.11)	0.5 (0.1 to 0.9)	1636/46 424	1.23 (1.11 to 1.36)	1.7 (0.8 to 2.6)	15.0
22.3 (21.9 to 22.6)	4590/115 784	1.15 (1.11 to 1.20)	1.1 (0.8 to 1.5)	1757/47 140	1.30 (1.17 to 1.43)	2.2 (1.2 to 3.1)	13.0
23.1 (22.6 to 23.5)	4747/111 929	1.27 (1.22 to 1.32)	2 (1.6 to 2.4)	1840/45 494	1.37 (1.24 to 1.51)	2.7 (1.7 to 3.7)	7.9
24.2 (23.5 to 25.1)	5459/113 753	1.47 (1.41 to 1.53)	3.4 (3 to 3.8)	2097/45 120	1.56 (1.41 to 1.73)	4.0 (2.9 to 5.1)	6.1
26.9 (25.1 to 54.7)^c^	7782/113 853	2.25 (2.17 to 2.33)	8.3 (7.7 to 8.8)	2878/43 799	2.10 (1.90 to 2.32)	7.2 (5.9 to 8.6)	−6.7
Cardiorespiratory fitness, W							
202 (100 to 211)^d^	10 149/131 756	Reference	Reference	3580/50 935	Reference	Reference	Reference
222 (212 to 229)^e^	8006/114 758	0.89 (0.86 to 0.91)	−1 (−1.2 to −0.7)	2978/45 822	0.98 (0.92 to 1.05)	−0.2 (−0.7 to 0.4)	−10.1
236 (230 to 241)	6518/108 462	0.86 (0.84 to 0.89)	−1.2 (−1.4 to −0.9)	2487/43 482	0.97 (0.90 to 1.04)	−0.2 (−0.8 to 0.3)	−12.8
250 (242 to 254)	5926/112 310	0.81 (0.79 to 0.84)	−1.5 (−1.8 to −1.3)	2233/46 088	0.91 (0.84 to 0.98)	−0.7 (−1.2 to −0.1)	−12.3
262 (255 to 270)	5175/124 210	0.77 (0.75 to 0.80)	−1.8 (−2.1 to −1.6)	2026/50 591	0.89 (0.82 to 0.97)	−0.8 (−1.4 to −0.3)	−15.6
277 (271 to 282)	3499/94 975	0.71 (0.68 to 0.74)	−2.4 (−2.7 to −2.2)	1449/39 526	0.90 (0.82 to 0.99)	−0.8 (−1.4 to −0.1)	−26.8
291 (283 to 300)	3317/119 447	0.70 (0.67 to 0.73)	−2.5 (−2.7 to −2.2)	1363/49 241	0.89 (0.81 to 0.98)	−0.8 (−1.5 to −0.2)	−27.1
311 (301 to 320)	2709/113 395	0.62 (0.59 to 0.65)	−3.1 (−3.4 to −2.9)	1098/47 700	0.79 (0.72 to 0.88)	−1.6 (−2.2 to −1.0)	−27.4
332 (321 to 343)	1751/107 738	0.57 (0.54 to 0.60)	−3.5 (−3.8 to −3.3)	713/44 119	0.76 (0.68 to 0.86)	−1.8 (−2.6 to −1.1)	−33.3
365 (344 to 999)	1556/111 782	0.55 (0.52 to 0.59)	−3.7 (−4.0 to −3.4)	671/46 491	0.77 (0.68 to 0.88)	−1.8 (−2.5 to −1.0)	−40.0
Systolic blood pressure, mm Hg							
112 (100 to 114)	4059/121 082	Reference	Reference	1580/49 626	Reference	Reference	Reference
120 (115 to 120)	7029/178 815	1.12 (1.07 to 1.16)	1 (0.6 to 1.3)	2632/73 310	1.04 (0.96 to 1.13)	0.3 (−0.4 to 1.0)	−7.1
122 (121 to 122)	2429/62 219	1.13 (1.07 to 1.19)	1.1 (0.7 to 1.6)	912/25 556	1.00 (0.89 to 1.11)	0.0 (−0.9 to 0.9)	−11.5
124 (123 to 126)	5924/149 474	1.14 (1.10 to 1.19)	1.2 (0.8 to 1.5)	2397/62 083	1.14 (1.04 to 1.24)	1.0 (0.3 to 1.8)	0.0
128 (127 to 128)	2765/63 448	1.23 (1.17 to 1.29)	1.9 (1.5 to 2.4)	1022/25 734	1.10 (0.99 to 1.22)	0.8 (−0.2 to 1.8)	−10.6
130 (129 to 132)	7276/171 911	1.26 (1.21 to 1.31)	2 (1.6 to 2.3)	2784/69 734	1.17 (1.07 to 1.27)	1.2 (0.4 to 1.9)	−7.1
134 (133 to 134)	2910/69 682	1.28 (1.22 to 1.34)	2.3 (1.8 to 2.8)	1076/28 634	1.13 (1.02 to 1.26)	1.1 (0.1 to 2.0)	−11.7
136 (135 to 138)	5004/109 636	1.38 (1.32 to 1.43)	2.9 (2.5 to 3.4)	1933/43 738	1.27 (1.15 to 1.39)	2.0 (1.1 to 2.9)	−8.0
140 (139 to 142)	5276/113 954	1.45 (1.39 to 1.51)	3.4 (3 to 3.9)	2038/45 285	1.29 (1.18 to 1.42)	2.1 (1.2 to 3.0)	−11.0
146 (143 to 180)	5923/98 612	1.67 (1.61 to 1.74)	5 (4.5 to 5.5)	2224/40 295	1.45 (1.32 to 1.60)	3.3 (2.2 to 4.3)	−13.2
Diastolic blood pressure, mm Hg							
50 (40 to 52)	2721/121 237	Reference	Reference	1203/53 619	Reference	Reference	Reference
56 (53 to 58)	4121/131 411	1.06 (1.00 to 1.11)	0.5 (0 to 0.9)	1738/57 051	1.06 (0.96 to 1.17)	0.5 (−0.3 to 1.3)	0.0
60 (59 to 60)	3439/107 811	1.07 (1.01 to 1.12)	0.6 (0.1 to 1)	1367/44 937	1.05 (0.95 to 1.16)	0.4 (−0.4 to 1.3)	−1.9
64 (61 to 64)	4698/126 055	1.13 (1.08 to 1.18)	1 (0.6 to 1.4)	1877/52 628	1.11 (1.01 to 1.22)	0.8 (0.0 to 1.7)	−1.8
68 (65 to 68)	6299/146 585	1.17 (1.12 to 1.23)	1.3 (0.9 to 1.7)	2423/59 384	1.15 (1.05 to 1.27)	1.1 (0.3 to 2.0)	−1.7
70 (69 to 70)	5723/128 012	1.23 (1.17 to 1.29)	1.8 (1.3 to 2.2)	2094/50 780	1.20 (1.09 to 1.32)	1.5 (0.6 to 2.4)	−2.4
72 (71 to 72)	2985/63 329	1.23 (1.17 to 1.30)	1.9 (1.4 to 2.4)	1168/25 439	1.22 (1.09 to 1.37)	1.7 (0.7 to 2.8)	−0.8
74 (73 to 76)	6181/118 216	1.29 (1.24 to 1.36)	2.2 (1.8 to 2.7)	2293/46 190	1.21 (1.10 to 1.33)	1.6 (0.7 to 2.5)	−6.2
80 (77 to 80)	6379/116 098	1.40 (1.33 to 1.46)	2.9 (2.4 to 3.4)	2320/44 364	1.30 (1.18 to 1.43)	2.2 (1.2 to 3.2)	−7.1
84 (81 to 100)	6060/80 079	1.70 (1.62 to 1.79)	5.2 (4.6 to 5.8)	2115/29 603	1.45 (1.31 to 1.61)	3.4 (2.2 to 4.5)	−14.7
Handgrip strength, N							
470 (100 to 500)	6259/136 462	Reference	Reference	2352/54 262	Reference	Reference	Reference
530 (501 to 540)	5638/126 176	0.93 (0.89 to 0.96)	−0.6 (−0.9 to −0.3)	2052/50 906	0.92 (0.85 to 1.00)	−0.6 (−1.3 to −0.0)	1.1
550 (541 to 560)	3439/85 206	0.89 (0.85 to 0.93)	−1 (−1.3 to −0.6)	1323/34 563	0.89 (0.83 to 1.05)	−0.9 (−1.6 to −0.3)	0.0
580 (561 to 590)	5980/135 760	0.90 (0.87 to 0.93)	−0.8 (−1.1 to −0.6)	2267/55 172	0.90 (0.83 to 0.98)	−0.8 (−1.4 to −0.2)	0.0
600 (591 to 610)	4175/102 543	0.89 (0.85 to 0.92)	−1 (−1.3 to −0.7)	1590/42 070	0.91 (0.84 to 1.00)	−0.7 (−1.4 to −0.1)	−2.2
630 (611 to 640)	5751/136 618	0.88 (0.85 to 0.91)	−1 (−1.2 to −0.7)	2157/55 617	0.89 (0.82 to 0.97)	−0.9 (−1.5 to −0.3)	−1.1
650 (641 to 660)	3269/82 770	0.87 (0.83 to 0.90)	−1.1 (−1.4 to −0.8)	1263/34 045	0.88 (0.80 to 0.97)	−1.0 (−1.7 to −0.3)	−1.1
680 (661 to 700)	5581/135 232	0.89 (0.85 to 0.92)	−0.9 (−1.2 to −0.7)	2192/55 348	0.97 (0.89 to 1.06)	−0.3 (−1.0 to 0.3)	−9.0
720 (701 to 740)	3832/87 776	0.90 (0.87 to 0.94)	−0.8 (−1.1 to −0.5)	1546/36 389	0.96 (0.87 to 1.06)	−0.4 (−1.1 to 0.3)	−6.7
780 (741 to 999)	4682/110 290	0.89 (0.85 to 0.92)	−1 (−1.2 to −0.7)	1856/45 623	0.90 (0.82 to 0.99)	−0.8 (−1.5 to −0.1)	−1.1
Combined risk, *z* score^f^							
−0.8 (−3.6 to −0.6)	1605/113 884	Reference	Reference	704/49 495	Reference	Reference	Reference
−0.5 (−0.6 to −0.4)	2362/113 883	1.20 (1.13 to 1.28)	1.7 (1.0 to 2.3)	996/48 699	1.12 (1.00 to 1.27)	1.0 (−0.1 to 2.1)	−6.7
−0.3 (−0.4 to −0.3)	2825/113 883	1.26 (1.19 to 1.35)	2.1 (1.5 to 2.7)	1187/48 313	1.16 (1.03 to 1.31)	1.3 (0.2 to 2.4)	−7.9
−0.2 (−0.3 to −0.1)	3293/113 884	1.33 (1.25 to 1.42)	2.6 (2.0 to 3.3)	1363/48 005	1.18 (1.05 to 1.33)	1.4 (0.3 to 2.4)	−11.3
−0.1 (−0.1 to 0)	4090/113 883	1.53 (1.44 to 1.62)	4.0 (3.4 to 4.7)	1620/47 242	1.28 (1.14 to 1.44)	2.1 (1.0 to 3.2)	−16.3
0 (0 to 0.1)	4566/113 883	1.60 (1.51 to 1.69)	4.5 (3.9 to 5.2)	1789/46 366	1.35 (1.20 to 1.52)	2.6 (1.4 to 3.8)	−15.6
0.2 (0.1 to 0.2)	5512/113 884	1.81 (1.71 to 1.92)	6.0 (5.2 to 6.7)	2172/45 940	1.54 (1.37 to 1.73)	3.9 (2.6 to 5.2)	−14.9
0.3 (0.2 to 0.4)	6169/113 883	1.92 (1.81 to 2.03)	6.7 (5.9 to 7.4)	2318/44 736	1.51 (1.34 to 1.69)	3.6 (2.4 to 4.9)	−21.4
0.5 (0.4 to 0.6)	7738/113 883	2.32 (2.20 to 2.45)	9.1 (8.3 to 10)	2799/43 449	1.72 (1.53 to 1.93)	5.0 (3.6 to 6.3)	−25.9
0.8 (0.6 to 4.2)^g^	10 446/113 883	3.12 (2.96 to 3.29)	13.6 (12.6 to 14.6)	3650/41 750	2.19 (1.96 to 2.46)	7.8 (6.2 to 9.4)	−29.8

Abbreviation: HR, hazard ratio.

^a^ The percentage change in HR was calculated as the difference in HRs over the cohort HR.

^b^ Body mass index is calculated as weight in kilograms divided by height in meters squared.

^c^ The maximum value observed in the tenth decile was 51.3 in the sibling cohort.

^d^ The first decile median in the sibling cohort was 203.

^e^ The second decile median in the sibling cohort was 223.

^f^ This is the unweighted mean across exposure *z* scores (see the Methods section).

^g^ The minimum observed value in the first decile was 3.7 in the sibling cohort.

### Cumulative CVD Incidence and PAF by Adolescent Risk Factors

Similarly, among the individual risk factors, cumulative CVD incidence varied markedly primarily by deciles of BMI, followed by blood pressure, whereas differences for cardiorespiratory fitness were smaller and those for handgrip strength were trivial (Table 2 and eFigure in Supplement 1). In the sibling analysis, the absolute incidence difference at age 60 years based on BMI was 1.5% (95% CI, 0.6%-2.3%) between the middle and bottom decile, increasing to 7.2% (95% CI, 5.9%-8.6%) between the top and bottom decile. In contrast, differences between levels of cardiorespiratory fitness remained at 0.8% or less up to the seventh decile, reaching 1.8% (95% CI, 1.0%-2.5%) in the top decile. Differences comparing the top vs bottom deciles were 3.3% (95% CI, 2.2%-4.3%) for systolic blood pressure, 3.4% (95% CI, 2.2%-4.5%) for diastolic blood pressure, 0.8% (95% CI, 0.1%-1.5%) for handgrip strength, and 7.8% (95% CI, 6.2%-9.4%) for the combined risk *z* score.

**Figure 2.**
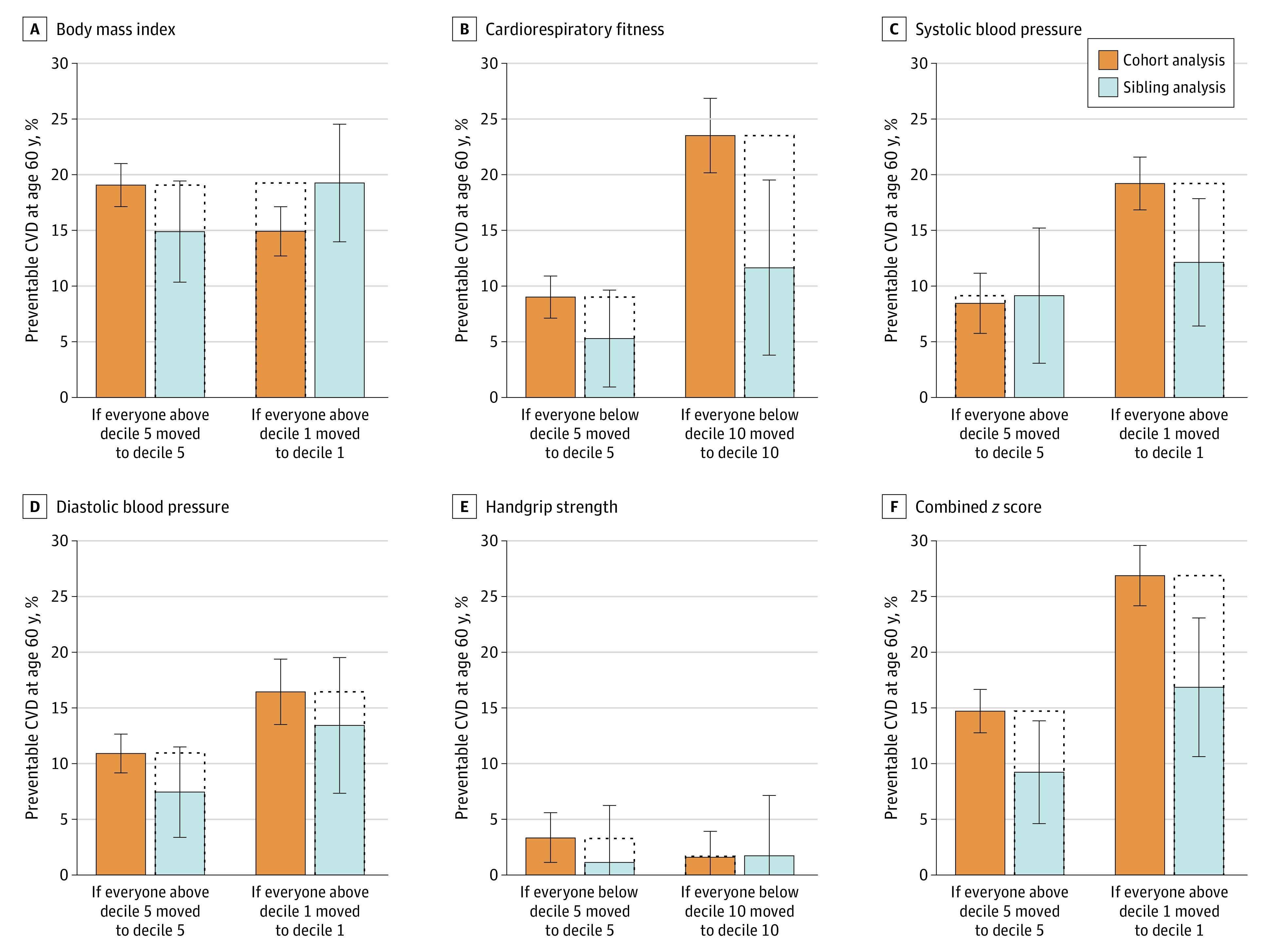
Population-Attributable Fraction for Cardiovascular Disease (CVD) by Risk Factors in the Total Cohort and Sibling Cohort, When Considering a Major or Moderate Intervention The major intervention entails shifting everyone to the best decile, whereas the moderate intervention involves shifting everyone from the worst to the middle decile. The combined *z* score represents the unweighted mean across exposure *z* scores (see the Methods section). Error bars indicate 95% CIs.

Accordingly, these incidence differences translated into a notable variation in the hypothetically preventable share of CVD at age 60 years (Figure 2), with BMI standing out as the risk factor with the highest PAF. The degree of attenuation between cohort and sibling analysis varied by risk factors and by whether one considered a major or a moderate intervention. For example, BMI was associated with a high PAF even with a moderate intervention, although it was slightly lower in the sibling analysis (14.9%) compared with the cohort analysis (19.1%). For a major intervention, the PAF associated with BMI was slightly higher in sibling analysis (19.3%) compared with cohort analysis (14.9%), as also reflected by the increased HRs associated with middle deciles in sibling analysis. In contrast, the attenuation observed for cardiorespiratory fitness translated into a clear decrease in the PAF, both when considering a major intervention (PAF, 23.5% vs 11.6%) and a moderate intervention (PAF, 9.0% vs 5.3%), consistent with the progressively attenuated HRs associated with deciles of cardiorespiratory fitness in sibling analysis. Interestingly, a moderate intervention on systolic blood pressure appeared largely unchanged in sibling analysis (PAF, 8.5% vs 9.1%). Finally, in the sibling analysis, the attenuation observed for the combined risk *z *score translated into a clear decrease in the PAF, both when considering a moderate intervention (PAF, 14.7% vs 9.2%) and a major intervention (PAF, 26.9% vs 16.9%).

## Discussion

This nationwide cohort study showed that cardiovascular risk factors in late adolescence were associated with future CVD and that controlling for unobserved genetic and environmental factors shared by full siblings altered the associations differently according to risk factors. The key findings were that a high BMI remained the most important factor associated with future CVD, whereas the benefits of cardiorespiratory fitness were more clearly attenuated.

A recent study^5^ reported an association between a combined risk *z* score in childhood and adolescence and CVD at approximately age 47 years. Our study indicates that a combined risk *z* score in late adolescence is associated with CVD even after controlling for shared familial factors, especially when estimated at age 60 years. We estimated that managing to shift everyone to the bottom decile of the combined risk *z* score through a major public health intervention could hypothetically prevent 16.9% of CVD events at age 60 years, after controlling for shared familial factors.

BMI stood out as the individual risk factor most associated with CVD, and the association was of similar magnitude as that of the combined risk *z* score. Unfortunately, other family-informed studies on BMI and CVD are rare; one twin study^18^ found an association between BMI and coronary heart disease mortality but not total CVD mortality, and another twin study^19^ found no association with myocardial infarction or all-cause mortality. Those studies were small, conducted in older populations, and differed in design and analytics, making comparisons with our study difficult. Although a dozen mendelian randomization studies render support for an effect of BMI on CVD,^30^ it has remained difficult to appreciate the magnitude of such an association. We find that even a modest improvement in adolescent BMI may have profound implications for CVD prevention, even after controlling for unobserved genetic and environmental factors shared by full siblings. Specifically, we estimate that managing to shift everyone from the poor spectra of BMI to the middle could hypothetically prevent 14.9% of CVD cases at age 60 years.

The relevance of a high BMI is reinforced as it also leads to elevated blood pressure,^30^ which we found to remain associated with CVD, despite some degree of attenuation in the sibling analysis. The fact that this was observed in a population of young men where the average blood pressure was within the normal range is worth highlighting, especially given the increasing prevalence of hypertension in this population during the last decades.^31^

In contrast, the benefits associated with high cardiorespiratory fitness were smaller and were more clearly attenuated in the sibling analysis. For example, the absolute difference in the CVD incidence at age 60 years across deciles of cardiorespiratory fitness was up to 4 times smaller than that seen for BMI. In addition, the proportion of CVD cases at age 60 that would be prevented if managing to shift all individuals to the top decile of fitness was reduced by half in the sibling analysis, from 23.5% to 11.6%. Although a PAF of 11.6% is clinically relevant, it is important to recognize that this estimate is based on a rather immense shift in the population’s cardiorespiratory fitness. These findings extend those from our previous study,^17^ where an attenuated association between adolescent cardiorespiratory fitness and a composite end point of CVD or all-cause mortality was observed in twins, but where the small number of cases hampered the certainty of the findings. Other large-scale observational studies on cardiorespiratory fitness and CVD have reported large associations, but these typically do not account for unobserved confounders.^9,10,11^ Our findings indicate that shared familial factors partly explain why fitter individuals have a lower risk of CVD and, by implication, that failing to account for such factors can result in inflated estimates. Such confounding by unmeasured familial factors may serve as one explanation for the conflicting findings between observational studies and randomized trials with respect to physical activity for CVD prevention.^32^

Regarding handgrip strength, we found that it had a trivial influence on CVD risk. Previous studies based on similar populations have also found beneficial, albeit often small, associations with cardiovascular outcomes.^7,11^ Our study adds new evidence by showing that the association appears similar after controlling for shared familial factors, but that the expected benefits of modifying muscular strength early in life (as proxied by handgrip) with respect to CVD risk seem small.

### Clinical Implications

Since 1975, global obesity rates in children and adolescents have quadrupled^33^ and are expected to continue increasing.^34^ Importantly, our findings suggest that even small improvements in BMI among adolescents could prevent a sizeable fraction of CVD cases later in life, even after controlling for shared familial factors, and despite having studied a relatively lean population. The opportunities for preventing CVD could be larger today, and further studies could be dedicated to projecting such prevention opportunities into the future.

Our findings also indicate that benefits of interventions targeting cardiorespiratory fitness in late adolescence do remain after controlling for unobserved confounders shared by full siblings. However, these benefits appear to be smaller than what is typically expected on the basis of conventional observational studies, and it seems as if quite substantial improvements in fitness levels are required to detect notable risk reductions. By implication, our findings suggest that public health interventions successfully targeting overweight and obesity may offer a greater net benefit to cardiovascular public health. Yet, on the basis of the totality of our findings, a broad, multifaceted public health intervention that aims to improve overall cardiovascular health among adolescents might still represent a promising approach, although most notably if it comes with positive effects on BMI.

### Limitations and Strengths

There are limitations to this work that should be kept in mind. First, although current evidence does not suggest different associations in women,^4^ it is important to recognize that only men were enrolled in this study. Second, although we calculated a combined risk *z* score based on cardiovascular risk factors that were available in the Swedish Military Service Conscription Registry, other important risk factors were not available, such as alcohol consumption, smoking status, lipid status, glycemic status, and diet. Third, as with any observational study, bias and confounding may be present, although the risk of reverse causation and selection bias by survival is arguably smaller in studies of youth compared with older people. Fourth, although sibling analysis is an effective way to account for all factors shared by siblings, we are unable to precisely pinpoint whether the attenuation observed is due to genetic or environmental confounding. It is possible that the attenuation is, in part, due to control for shared socioeconomic influences, although our previous work does not support a large role of such factors in the link between adolescent cardiorespiratory fitness and future CVD.^17^ Fifth, because our sibling analysis accounted for only approximately 50% of the genetic makeup of an individual (ie, the genetic proportion shared by full siblings), it may not have accounted for all the genes of importance to the risk factors and outcome. Residual confounding, particularly from nonshared genes, therefore, likely remains. Sixth, although sibling analysis is a powerful tool to adjust for confounding factors shared within families, it rests on several assumptions.^20^ For example, inflation of measurement error and control for shared mediators can bias sibling analysis toward the null. If such processes were present in our study, they could partly explain the observed attenuation.

However, this work has several major strengths. For instance, leveraging data from multiple nationwide registers to perform sibling comparisons allowed us to better assess the nature of the association of cardiovascular risk factors in late adolescence with future CVD, compared with conventional cohort studies. Furthermore, our sibling analysis comprised almost half a million full brothers and more than 18 000 CVD events, meaning that it had substantial statistical power. Other strengths include a comprehensive set of analyses, the long follow-up, and the ascertainment of CVD using nationwide health registries and death certificates with high precision. Finally, the use of registries in a universal health care system virtually eliminated the loss to follow-up, apart from the 6.3% of participants who emigrated out of Sweden. Together, these features strengthen the validity of our findings.

## Conclusions

In this Swedish national cohort study, cardiovascular risk factors in late adolescence were associated with CVD in adulthood. After using sibling analysis to control for unobserved genetic and shared environmental factors, a high BMI was the primary individual risk factor, whereas the benefits of cardiorespiratory fitness were more clearly attenuated. For effective CVD prevention, these findings suggest that the priority among policymakers and public health authorities should be to aim the efforts toward halting and reversing the obesity epidemic.

## Notes

Supplement 1. **eTable 1.** List of Diagnostic Codes for the Outcomes in the Study **eFigure.** Cumulative Cardiovascular Disease Incidence by Deciles of Risk Factors in the Total Cohort and Sibling Cohort **eTable 2.** Hazard Ratios for Cardiovascular Disease by Tenths (Deciles) of the Individual Risk Factors and the Combined Risk Z-Score in the Total Cohort (as Reported in the Main Article) When Replicating Standard Analysis in the Sibling Cohort, and Sibling Analysis (as Reported in the Main Article)

Supplement 2.
Data Sharing Statement
