# Supplementary material for: Genetic and Environmental Factors and Cardiovascular Disease Risk in Adolescents
Source: JAMA Netw Open. 2023 Nov 17;6(11):e2343947. doi: 10.1001/jamanetworkopen.2023.43947 (PMC10656641; doi:10.1001/jamanetworkopen.2023.43947)
Supplement: Supplement 1. — eTable 1. List of Diagnostic Codes for the Outcomes in the Study eFigure. Cumulative Cardiovascular Disease Incidence by Deciles of Risk Factors in the Total Cohort and Sibling Cohort eTable 2. Hazard Ratios for Cardiovascular Disease by Tenths (Deciles) of the Individual Risk Factors and the Combined Risk Z-Score in the Total Cohort (as Reported in the Main Article) When Replicating Standard Analysis in the Sibling Cohort, and Sibling Analysis (as Reported in the Main Article) [file jamanetwopen-e2343947-s001.pdf]

# Supplemental Online Content

Ballin M, Neovius M, Ortega FB. Genetic and environmental factors and cardiovascular disease risk in adolescents. *JAMA Netw Open*. 2023;6(11):e2343947.
doi:10.1001/jamanetworkopen.2023.43947

**eTable 1.** List of Diagnostic Codes for the Outcomes in the Study
**eFigure.** Cumulative Cardiovascular Disease Incidence by Deciles of Risk Factors in the Total Cohort and Sibling Cohort
**eTable 2.** Hazard Ratios for Cardiovascular Disease by Tenths (Deciles) of the Individual Risk Factors and the Combined Risk Z-Score in the Total Cohort (as Reported in the Main Article) When Replicating Standard Analysis in the Sibling Cohort, and Sibling Analysis (as Reported in the Main Article)

This supplemental material has been provided by the authors to give readers additional information about their work.

© 2023 Ballin M et al. *JAMA Network Open.*

**eTable 1. List of Diagnostic Codes for the Outcomes in the Study**

| Variable | Code Type | Codes |
|---|---|---|
| Death from cardiovascular causes | ICD-10 | I00-I99 |
| | ICD-9 | 390-459 |
| | ICD-8 | 390-458 |
| Ischemic heart disease | ICD-10 | I20-I25 |
| | ICD-9 | 410-414 |
| | ICD-8 | 410-414 |
| Stroke | ICD-10 | I60, I61, I63, I64 |
| | ICD-9 | 430-438 |
| | ICD-8 | 430-438 |
| Heart failure | ICD-10 | I50 |
| | ICD-9 | 428A, 428B, 428X |
| | ICD-8 | -* |

*Heart failure using ICD-8 could not be tracked as we did not have access to four-digit codes. However, we deem the number of such cases to have been negligible given that most of the years of follow-up were covered using ICD-9 and 10, and because heart failure is very uncommon at an age corresponding to the age of the participants when ICD-8 was still in use.

© 2023 Ballin M et al. *JAMA Network Open.*

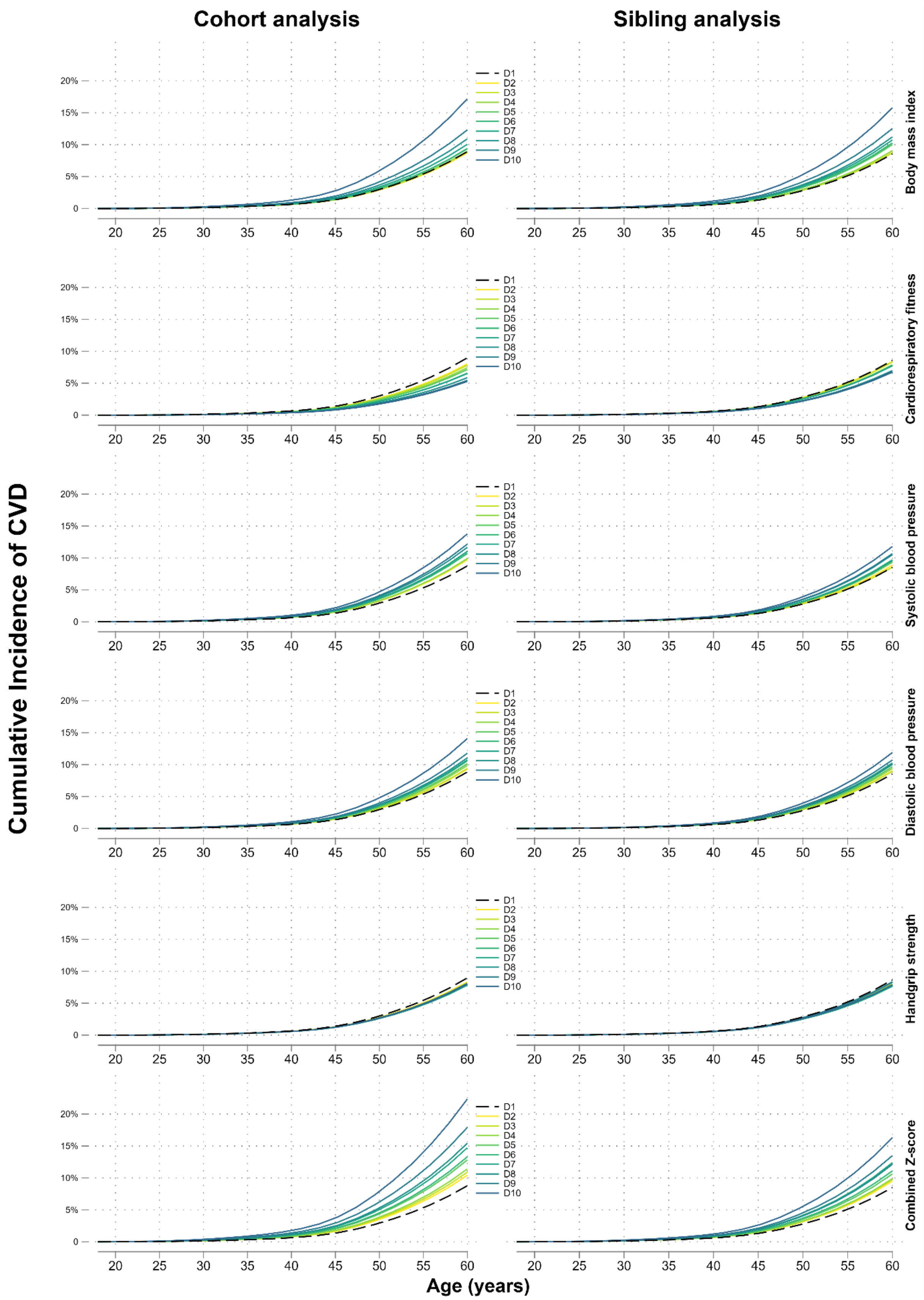


**eFigure. Cumulative Cardiovascular Disease Incidence by Deciles of Risk Factors in the Total Cohort and Sibling Cohort.** The underlying timescale is the time since conscription, but for ease of interpretation, we re-label the x-axis to age as 92.2% conscripted at age 18. Estimates represent the 1-Survival obtained using flexible parametric survival models. Inferential measures are omitted for clarity as they are also reflected in Figure 1 and Table 2. The combined Z-score represents the unweighted mean across exposure Z-scores (see method section). CVD = cardiovascular disease.

© 2023 Ballin M et al. *JAMA Network Open.*

**eTable 2. Hazard Ratios for Cardiovascular Disease by Tenths (Deciles) of the Individual Risk Factors and the Combined Risk Z-Score in the Total Cohort (as Reported in the Main Article) When Replicating Standard Analysis in the Sibling Cohort, and Sibling Analysis (as Reported in the Main Article).**

| | Cohort analysis (N=1,138,833) (as reported in main article) | Standard analysis replicated in sibling cohort (N=463,995) | Sibling analysis (N=463,995) (as reported in main article) |
|---|---|---|---|
| **Risk factor value per decile, median (range)** | **HR (95% CI)** | **HR (95% CI)** | **HR (95% CI)** |
| **Body mass index, kg/m$^2$** | | | |
| 18.1 (15.0, 18.7) | Ref. | Ref. | Ref. |
| 19.2 (18.7, 19.6) | 0.98 (0.94, 1.02) | 1.03 (0.96, 1.1) | 1.06 (0.97, 1.16) |
| 19.9 (19.6, 20.2) | 1.00 (0.96, 1.05) | 1.01 (0.94, 1.08) | 1.02 (0.93, 1.12) |
| 20.5 (20.2, 20.8) | 1.01 (0.97, 1.05) | 1.02 (0.95, 1.09) | 1.06 (0.97, 1.17) |
| 21.1 (20.8, 21.3) | 1.06 (1.02, 1.10) | 1.09 (1.02, 1.17) | 1.19 (1.08, 1.31) |
| 21.6 (21.3, 21.9) | 1.07 (1.02, 1.11) | 1.09 (1.02, 1.17) | 1.23 (1.11, 1.36) |
| 22.3 (21.9, 22.6) | 1.15 (1.11, 1.20) | 1.18 (1.1, 1.26) | 1.30 (1.17, 1.43) |
| 23.1 (22.6, 23.5) | 1.27 (1.22, 1.32) | 1.31 (1.23, 1.4) | 1.37 (1.24, 1.51) |
| 24.2 (23.5, 25.1) | 1.47 (1.41, 1.53) | 1.54 (1.45, 1.65) | 1.56 (1.41, 1.73) |
| 26.9 (25.1, 54.7)[a] | 2.25 (2.17, 2.33) | 2.34 (2.21, 2.49) | 2.10 (1.90, 2.32) |
| **Cardiorespiratory fitness, watt** | | | |
| 202 (100, 211)[b] | Ref. | Ref. | Ref. |
| 222 (212, 229)[c] | 0.89 (0.86, 0.91) | 0.91 (0.86, 0.95) | 0.98 (0.92, 1.05) |
| 236 (230, 241) | 0.86 (0.84, 0.89) | 0.89 (0.85, 0.94) | 0.97 (0.90, 1.04) |
| 250 (242, 254) | 0.81 (0.79, 0.84) | 0.8 (0.76, 0.85) | 0.91 (0.84, 0.98) |
| 262 (255, 270) | 0.77 (0.75, 0.80) | 0.78 (0.74, 0.82) | 0.89 (0.82, 0.97) |
| 277 (271, 282) | 0.71 (0.68, 0.74) | 0.74 (0.69, 0.79) | 0.90 (0.82, 0.99) |
| 291 (283, 300) | 0.70 (0.67, 0.73) | 0.7 (0.66, 0.75) | 0.89 (0.81, 0.98) |
| 311 (301, 320) | 0.62 (0.59, 0.65) | 0.6 (0.56, 0.64) | 0.79 (0.72, 0.88) |
| 332 (321, 343) | 0.57 (0.54, 0.60) | 0.56 (0.51, 0.6) | 0.76 (0.68, 0.86) |
| 365 (344, 999) | 0.55 (0.52, 0.59) | 0.56 (0.51, 0.61) | 0.77 (0.68, 0.88) |
| **Systolic blood pressure, mmHg** | | | |
| 112 (100, 114) | Ref. | Ref. | Ref. |
| 120 (115, 120) | 1.12 (1.07, 1.16) | 1.07 (1.01, 1.14) | 1.04 (0.96, 1.13) |
| 122 (121, 122) | 1.13 (1.07, 1.19) | 1.07 (0.99, 1.16) | 1.00 (0.89, 1.11) |
| 124 (123, 126) | 1.14 (1.10, 1.19) | 1.18 (1.11, 1.26) | 1.14 (1.04, 1.24) |
| 128 (127, 128) | 1.23 (1.17, 1.29) | 1.17 (1.08, 1.27) | 1.10 (0.99, 1.22) |
| 130 (129, 132) | 1.26 (1.21, 1.31) | 1.25 (1.17, 1.33) | 1.17 (1.07, 1.27) |
| 134 (133, 134) | 1.28 (1.22, 1.34) | 1.21 (1.12, 1.31) | 1.13 (1.02, 1.26) |
| 136 (135, 138) | 1.38 (1.32, 1.43) | 1.41 (1.32, 1.5) | 1.27 (1.15, 1.39) |
| 140 (139, 142) | 1.45 (1.39, 1.51) | 1.47 (1.37, 1.57) | 1.29 (1.18, 1.42) |
| 146 (143, 180) | 1.67 (1.61, 1.74) | 1.65 (1.55, 1.77) | 1.45 (1.32, 1.60) |
| **Diastolic blood pressure, mmHg** | | | |
| 50 (40, 52) | Ref. | Ref. | Ref. |
| 56 (53, 58) | 1.06 (1.00, 1.11) | 1.06 (0.98, 1.14) | 1.06 (0.96, 1.17) |

© 2023 Ballin M et al. *JAMA Network Open.*

| | | | |
|---|---|---|---|
| 60 (59, 60) | 1.07 (1.01, 1.12) | 1.07 (0.99, 1.16) | 1.05 (0.95, 1.16) |
| 64 (61, 64) | 1.13 (1.08, 1.18) | 1.14 (1.05, 1.22) | 1.11 (1.01, 1.22) |
| 68 (65, 68) | 1.17 (1.12, 1.23) | 1.19 (1.11, 1.28) | 1.15 (1.05, 1.27) |
| 70 (69, 70) | 1.23 (1.17, 1.29) | 1.23 (1.15, 1.33) | 1.20 (1.09, 1.32) |
| 72 (71, 72) | 1.23 (1.17, 1.30) | 1.29 (1.19, 1.4) | 1.22 (1.09, 1.37) |
| 74 (73, 76) | 1.29 (1.24, 1.36) | 1.33 (1.23, 1.43) | 1.21 (1.10, 1.33) |
| 80 (77, 80) | 1.40 (1.33, 1.46) | 1.44 (1.34, 1.54) | 1.30 (1.18, 1.43) |
| 84 (81, 100) | 1.70 (1.62, 1.79) | 1.73 (1.6, 1.86) | 1.45 (1.31, 1.61) |
| **Handgrip strength, newton** | | | |
| 470 (100, 500) | Ref. | Ref. | Ref. |
| 530 (501, 540) | 0.93 (0.89, 0.96) | 0.89 (0.84, 0.95) | 0.92 (0.85, 1.00) |
| 550 (541, 560) | 0.89 (0.85, 0.93) | 0.89 (0.84, 0.96) | 0.89 (0.83, 1.05) |
| 580 (561, 590) | 0.90 (0.87, 0.93) | 0.89 (0.84, 0.95) | 0.90 (0.83, 0.98) |
| 600 (591, 610) | 0.89 (0.85, 0.92) | 0.88 (0.82, 0.93) | 0.91 (0.84, 1.00) |
| 630 (611, 640) | 0.88 (0.85, 0.91) | 0.86 (0.82, 0.92) | 0.89 (0.82, 0.97) |
| 650 (641, 660) | 0.87 (0.83, 0.90) | 0.86 (0.81, 0.92) | 0.88 (0.80, 0.97) |
| 680 (661, 700) | 0.89 (0.85, 0.92) | 0.9 (0.85, 0.95) | 0.97 (0.89, 1.06) |
| 720 (701, 740) | 0.90 (0.87, 0.94) | 0.94 (0.88, 1) | 0.96 (0.87, 1.06) |
| 780 (741, 999) | 0.89 (0.85, 0.92) | 0.9 (0.85, 0.96) | 0.90 (0.82, 0.99) |
| **Combined risk, Z-score*** | | | |
| -0.8 (-3.6, -0.6) | Ref. | Ref. | Ref. |
| -0.5 (-0.6, -0.4) | 1.20 (1.13, 1.28) | 1.19 (1.08, 1.31) | 1.12 (1.00, 1.27) |
| -0.3 (-0.4, -0.3) | 1.26 (1.19, 1.35) | 1.27 (1.16, 1.39) | 1.16 (1.03, 1.31) |
| -0.2 (-0.3, -0.1) | 1.33 (1.25, 1.42) | 1.34 (1.22, 1.47) | 1.18 (1.05, 1.33) |
| -0.1 (-0.1, 0) | 1.53 (1.44, 1.62) | 1.51 (1.38, 1.65) | 1.28 (1.14, 1.44) |
| 0 (0, 0.1) | 1.60 (1.51, 1.69) | 1.60 (1.47, 1.75) | 1.35 (1.20, 1.52) |
| 0.2 (0.1, 0.2) | 1.81 (1.71, 1.92) | 1.86 (1.7, 2.02) | 1.54 (1.37, 1.73) |
| 0.3 (0.2, 0.4) | 1.92 (1.81, 2.03) | 1.94 (1.78, 2.11) | 1.51 (1.34, 1.69) |
| 0.5 (0.4, 0.6) | 2.32 (2.20, 2.45) | 2.32 (2.14, 2.53) | 1.72 (1.53, 1.93) |
| 0.8 (0.6, 4.2)[d] | 3.12 (2.96, 3.29) | 3.19 (2.94, 3.46) | 2.19 (1.96, 2.46) |

© 2023 Ballin M et al. *JAMA Network Open.*
