# Supplementary material for: Genetic and Environmental Factors and Cardiovascular Disease Risk in Adolescents
Source: JAMA Netw Open. 2023 Nov 17;6(11):e2343947. doi: 10.1001/jamanetworkopen.2023.43947 (PMC10656641; doi:10.1001/jamanetworkopen.2023.43947)
Supplement: Supplement 2. — Data Sharing Statement [file jamanetwopen-e2343947-s002.pdf]

Data Sharing Statement

Ballin. Genetic and Environmental Factors and Cardiovascular Disease Risk in Adolescents. *JAMA Netw Open*. Published November 17, 2023. doi:10.1001/jamanetworkopen.2023.43947

Data
Data available: No
